# Never too late to start musical instrument training: Effects on working memory and subcortical preservation in healthy older adults across 4 years

**DOI:** 10.1162/IMAG.a.48

**Published:** 2025-06-17

**Authors:** Xueyan Wang, Masatoshi Yamashita, Xia Guo, Lars Stiernman, Marcelo Kakihara, Nobuhito Abe, Kaoru Sekiyama

**Affiliations:** Graduate School of Advanced Integrated Studies in Human Survivability, Kyoto University, Kyoto, Japan; Graduate School of Social and Cultural Sciences, Kumamoto University, Kumamoto, Japan; Department of Medical and Translational Biology, Umeå University, Umeå, Sweden; Umeå Center for Functional Brain Imaging, Umeå University, Umeå, Sweden; Institute for the Future of Human Society, Kyoto University, Kyoto, Japan; Sichuan Provincial People’s Hospital, Chengdu, China; University of Electronic Science and Technology of China, Chengdu, China; Research Center for Child Mental Development, University of Fukui, Fukui, Japan; School of Humanities and Social Sciences, Shanxi Medical University, Shanxi, China; Research Center for Psychological and Health Sciences, Shanxi Medical University, Shanxi, China; Wildlife Research Center, Kyoto University, Kyoto, Japan

**Keywords:** verbal working memory, putamen, cerebellum, musical instrument training, healthy aging

## Abstract

Studies have shown the beneficial effects of musical instrument on memory and executive function in healthy aging. However, few studies investigated these long-term benefits. In this regard, the current study tracked a cohort of older adults (n = 53) over 4 years after they have initially participated in a musical instrument training program. Out of the initial sample, 13 of them voluntarily continued participating in the musical instrument training (continue group: 77.85 ± 4.30 years, 10 female, 3 male), while 19 of them discontinued their participation in the music program and instead engaged in other forms of leisure activities (stop group: age: 76.00 ± 5.44 years, 13 female, 6 male). At baseline, behavioral measures of verbal working memory (WM), verbal memory, and executive control were collected. In addition, participants completed a visual WM task (face n-back task) during fMRI scanning. Four years later, the same battery of tests was administered, with the addition of a digit n-back task to examine changes in verbal WM. Region-of-interest structural analyses focused on the striatum and cerebellum, based on previously reported intervention effects and the advantages observed in musicians. The continue group demonstrated better preservation of verbal WM performance (a composite score of Digit Span and Verbal Fluency tasks) and right putamen gray matter volume (GMV) over 4 years. During verbal WM processing, this group exhibited lower cerebellum–pons functional connectivity (FC), which significantly correlated with improved verbal WM performance. Moreover, the continue group also showed greater cerebellar activation during the digit task, increased intra-cerebellar FC, and decreased cerebellar–cortical FC during the face task. The combined evidence suggested enhanced cerebellar function and thus reduced reliance on other brain regions such as the cortical areas and brainstem for compensation. Taken together, these results suggested the musical instrument training effects in mitigating age-related decline in verbal WM and subcortical structure (putamen) and function (cerebellum). This study provides longitudinal evidence that initiating musical instrument training in older adulthood can counteract age-related cognitive and brain decline.

## 1 Introduction

Memory decline, especially decline in episodic memory and working memory (WM), is a significant characteristic of normal aging (Hultsch et al., 1992;Nyberg et al., 2012;Park et al., 2002), and can be an early sign of pathological aging (Gold & Budson, 2008). However, older musicians show less decline in memory compared with age-matched non-musicians (seeTable 1;Böttcher et al., 2022;Fauvel et al., 2014;Gray & Gow, 2020;Hanna-Pladdy & Gajewski, 2012;Hanna-Pladdy & MacKay, 2011;Mansens et al., 2018;Vetere et al., 2024;Yamashita et al., 2022), and musical instrument training has been linked to lower risk of cognitive impairment in older adults (Mansky et al., 2020;Walsh et al., 2021). In light of these promising behavioral observations, existing studies have started to document the neural correlates that could be involved in the beneficial effects of musical instrument training on the preservation of memory functioning in older adults.

**Table 1.** Memory-related measures where cross-sectional studies revealed advantages in older musicians compared with age-matched non-musicians.

Study	Memory assessments	Memory domains
Böttcher et al., 2022	Digit Span Forward and Backward, Free and Cued Selective Reminding Test interference task (Serial 3s)	WM
Fauvel et al., 2014	Digit Span Forward, Phonological fluency, Semantic fluency.	WM
Gray & Gow, 2020	Digit Span Forward and Backward.	WM
Hanna-Pladdy & Gajewski, 2012	Phonological Fluency, California Verbal Learning Test second version short delay free recall.	WM, EM
Hanna-Pladdy & MacKay, 2011	Nonverbal memory recall of Visual Reproduction, Boston Naming Test.	WM, EM
Mansens et al., 2018	Phonological Fluency, Digit Span Forward and Backward, Auditory Verbal Learning Test.	WM, EM
Vetere et al., 2024	Paired Associate Learning, Digit Span, Self-Ordered Search.	WM
Yamashita et al., 2022	Semantic Fluency.	WM

WM, working memory; EM, episodic memory.

Better cognitive performance has been linked to fewer age-related reductions in brain volume (Johansson et al., 2022;Nyberg et al., 2022). Longitudinal studies have revealed rapid declines in subcortical structures including striatum and the cerebellum among healthy older adults (Bagarinao et al., 2022;Fjell et al., 2009;Park & Reuter-Lorenz, 2009;Raz et al., 2010). In contrast, older instrumental musicians have demonstrated greater gray matter volume (GMV) in cerebellum than age-matched non-musicians (Böttcher et al., 2022;Yamashita et al., 2022). In addition to the structural advantage, musicians with lifelong instrumental training experience have shown greater cerebellar–hippocampal functional connectivity (FC) during melodic WM processing, reflecting stronger subcortical network coupling (Yamashita et al., 2022).

Longitudinal evidence has suggested that the neural advantages related to musical instrument training may result from an accumulated reserve built up across different life phases. Musical instrument training interventions in school-aged children improved their language ability (Barbaroux et al., 2019;Rickard et al., 2010), WM (Barbaroux et al., 2019;Guo et al., 2018;Rickard et al., 2010;Roden, Grube, et al., 2014;Roden, Könen, et al., 2014) and attention control (Hennessy et al., 2019;Roden, Könen, et al., 2014). In young musicians compared with non-musicians, functional neuroimaging studies have revealed lower activation in prefrontal cortex (PFC), reflecting increased efficiency, (Alain et al., 2018), and better task performance and larger BOLD response in the right putamen during auditory WM processing, reflecting an increased ability to recruit neural resources (Pallesen et al., 2010). Additionally, greater GMV in the basal ganglia and cerebellum has been observed in young musicians, suggestive of accelerated development related to early-age musical instrument training (Acer et al., 2018;Gaser & Schlaug, 2003;Vaquero et al., 2016). Short-term musical instrument training interventions have improved episodic memory, WM, and executive control in healthy older adults (Bugos et al., 2007;Guo et al., 2021;Wang et al., 2023), suggesting that that cognitive reserves continue to accumulate in older age with musical instrument training.

In a previous study including the present sample, participants showed decreased FC between the left putamen and the right superior temporal gyrus during a visual WM task, which correlated with greater improvement in verbal memory following a 16-week melodica intervention (Guo et al., 2021). These results indicate that musical instrument training enhances neural ability in the putamen aiding WM processing. Additionally,Marie et al. (2023)reported an increase in the cerebellar GMV after a 6-month musical instrument training intervention in older adults, linked to enhanced tonal WM processing. Combining these findings with evidence showing benefits to brain health in older musicians with lifelong musical instrument training experience, it is suggested that musical instrument training contributes to memory enhancement through the effects on the structure and function of striatum and cerebellum. This might be due to the critical role of the striatum and cerebellum in temporal information processing in WM (Teki & Griffiths, 2016), and motor sequence learning (Bednark et al., 2015;Koeneke et al., 2004;Penhune & Steele, 2012). Furthermore, a recent review byHeng et al. (2024)highlighted the potential of Bayesian inference as a fundamental principle for explaining the effects of music-based interventions on aging, emphasizing the involvement of the striatum and cerebellum in the Bayesian brain mechanisms associated with music making. Activities of music making require precise beat synchronization, motor control, and WM for music phrases, likely strengthening the striatum and cerebellum through repeated sensory coordination and action planning and selection during music making.

To summarize, cross-sectional comparison between older musicians and age-matched non-musicians has shown an association between musical instrument training experience and better cognitive performance and brain reserve (Böttcher et al., 2022;Fauvel et al., 2014;Gray & Gow, 2020;Hanna-Pladdy & Gajewski, 2012;Hanna-Pladdy & MacKay, 2011;Mansens et al., 2018;Vetere et al., 2024;Yamashita et al., 2022). However, the association cannot be solely attributed to musical instrument training. Musicians’ cognitive advantages may stem from pre-existing characteristics (Román-Caballero et al., 2022), such as personality traits (Corrigall et al., 2013) or music aptitude (Swaminathan et al., 2017), prior to the onset of training. Furthermore, the cross-sectional design of these studies limited their ability to infer causation. In response, randomized controlled trial interventions have been conducted, demonstrating that musical instrument training for 3 to 6 months causally improved cognition and neural efficiency in musically untrained older adults (Bugos et al., 2007;Guo et al., 2021;Wang et al., 2023). However, due to the nature of the study design, the cognitive benefits induced by the short-term programs might also be due to the environmental novelty rather than musical instrument training itself. Moreover, short intervals between tests are not suitable for investigating whether musical instrument training can mitigate age-related cognitive decline which is observed over several years.

Therefore, to clarify the musical instrument training effects on mitigating age-related cognitive decline, we monitored neurocognitive changes across 4 years in a group of healthy older adults who either continued musical instrument training or discontinued after 12 to 16 weeks of participation of a musical instrument training program (Guo et al., 2021). In the original study, participants in the intervention group completed 16 weeks of instrument training. Participants in the control group was provided with 12 weeks of instrument training after the study was over, as promised during recruitment. We tracked the changes in WM, episodic memory, and executive control, based on previously reported short-term intervention effects (Bugos et al., 2007;Guo et al., 2021;Wang et al., 2023). Additionally, building on the previous research, we hypothesize that the preservation of striatal and cerebellar structure and function mediates the positive musical instrument training effects on behavioral outcomes.

## 2 Method

### 2.1 Participants

Recruitment was conducted among participants from our previous musical instrument training program for musically untrained healthy older adults held at Kyoto City Sakyo Elderly Welfare Center, Japan (Guo et al., 2021). Participants were excluded based on the baseline tests conducted in January to February 2018, which included the Mini-Mental State Examination (MMSE) and Wechsler Memory Scale-Revised Logical Memory delayed recall (LM-II), brain imaging results, and self-report psychiatric disorder history and musical experience. Exclusion criteria at baseline included (1) evidence of brain damage on the structural MRI; (2) a history of neurological or psychiatric disorders; (3) an MMSE score below 25; (4) an LM-II score more than 2 standard deviations below their age-appropriate means; (5) withdrawal due to health issues during the intervention program; (6) more than 3 years of musical instrument training in the past or any training in the last 5 years. All participants were right handed according to a self-report questionnaire.

Fifty-three participants initially joined the program. Participants in the intervention group were initially scheduled for a 12-week training program. However, due to a malfunction of the MRI equipment during the 13th to 16th weeks, these participants received additional 4-week training sessions beyond the planned duration. As a result, the intervention group completed 16 weeks of training from March to July 2018. In contrast, participants in the control group completed the scheduled 12 sessions from September to December 2018, after post-waiting tests. The intervention program provided instructions on reading basic music scores, exercises to improve finger dexterity and independence, and progressively challenging musical performance tasks. By the end of the program, participants could read the notes and their corresponding fingering, understand the relationship between notes at G clef and keys on the melodica, note and rest durations, and the relationship between air and sound articulation in the melodica. They successfully mastered playing 11 songs. After the initial program was over, some participants voluntarily continued melodica training, while others quit musical instrument training but continued other leisure activities, such as table tennis, gymnastics, and stretching exercise in the center.

The continue group started a melodica club at the center. They were motivated by opportunities to perform at public concerts held at least twice a year: one in July for the Summer Tanabata festival and another in December before Christmas. They participated in group training once a week: a 1-hour session on bi-monthly Saturday mornings with all club members and a 2-hour session on bi-monthly Monday mornings where they practiced in a smaller class either as the early or late morning group. In addition to group training, they also practiced at home. Due to the center’s closure during the COVID-19 pandemic, the group training was ceased from April to May 2020 and again from August to September 2021.

During the intervention and subsequent practice until the pandemic, the group used the Yamaha P-32EP melodica for group training at the center. Starting in November 2021, they switched to the Yamaha Remie PSS-E30 (electric keyboards) for group sessions to reduce the risk of the COVID-19 infection. However, throughout the entire period, they continued practicing individually at home using the original Yamaha P-32EP melodica. Group training sessions included technique exercises, where participants played sections or the full C scale in ascending and descending movements using all fingers of the right hand; warm-ups, which involved stretching the body, hands, and fingers; individual practice, where participants sang the melody using note names and lyrics (when applicable) while playing sections of tunes; and ensemble playing, where they performed tunes in unison by the teacher’s piano accompaniment.

Ultimately, as shown inFigure 1, a total of 32 healthy older adults (23 women; n = 13 in the continue group, n = 19 in the stop group) participated in this study from December 2021 to February 2022, approximately 4 years after the baseline (mean test interval = 3.96 years, SD = 0.16 years). Before the follow-up assessment, participants were tested two more times after the baseline: once in May or July 2018 and again from August to September 2018. Other potential participants ceased attending the center or were unable to participate in tests due to personal health reasons. Participants in the follow-up assessment (Participant Group: PG) and those who drop out (Drop-out Group: DG) showed no statistically significant difference in age (*p*= 0.678, PG: mean = 72.81, SD = 4.97; DG: mean = 73.46, SD = 6.30 years at baseline), years of education (*p*= 0.063, PG: mean = 13.66, SD = 2.01; DG: mean = 12.68, SD = 1.59), and baseline MMSE (*p*= 0.388, PG: mean = 28.69, SD = 1.06; DG: mean = 28.36, SD = 1.68). The protocol was approved by the ethics committee of Kyoto University (3-P-14). All participants provided written informed consent.

**Fig. 1.**
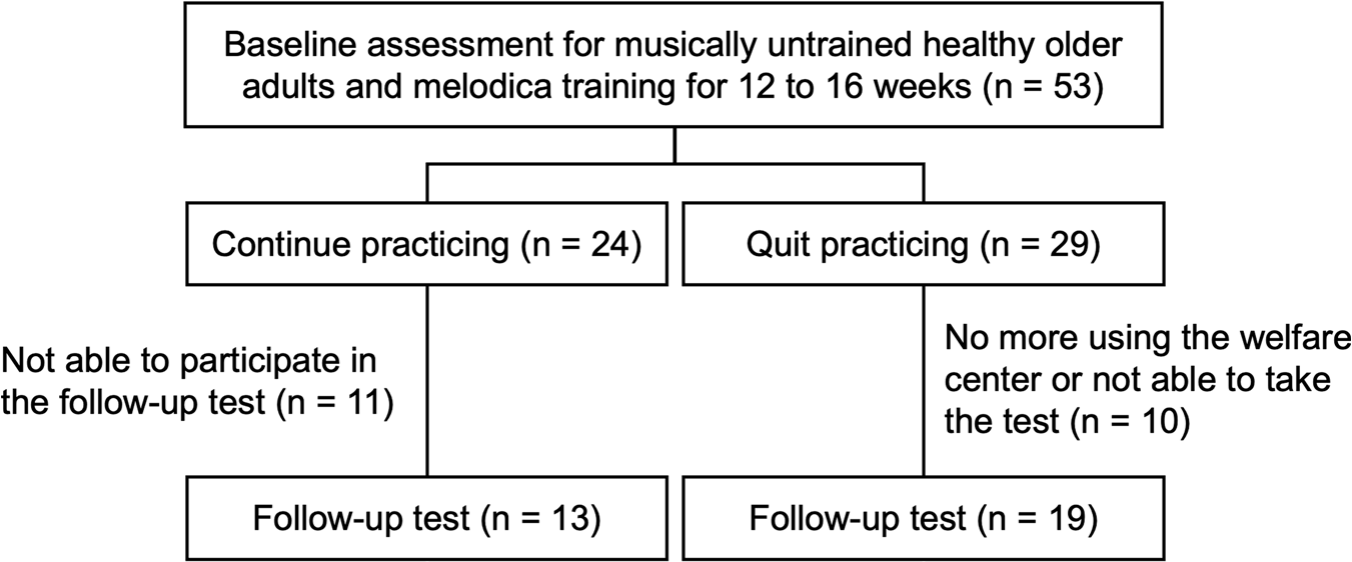
Distribution of participants.

### 2.2 Behavioral tests

To confirm whether these two groups were homogenous at baseline, we conducted an independent two-sample t-test for the demographic characteristics and baseline behavioral performance and striatal and cerebellar GMV. To assess longitudinal changes, we repeated the same neuropsychological assessments used in the baseline test of our previous short-term intervention study. The examiners who carried out the neuropsychological assessments were blind to the membership of participants in the two groups.

We measured verbal WM using the composite score of Digit Span and Verbal Fluency Test (Lezak et al., 2004;Richardson, 2007). We calculated the mean and standard deviation of the baseline scores for the continue group and stop group, treating them as a single pooled sample. Next, we computed the Z score for each participant using the formula Z = (x – M)/SD, where x is the participant’s raw score. Positive Z-values indicate scores above the pooled sample mean, while negative values indicate below-average score. Z scores were calculated for each participant across phonological and semantic verbal fluency, Digit Span forward and backward, then averaged to create a composite WM score. Episodic memory was assessed using the same procedures as the Logical Memory Test as at baseline (Sugishita et al., 2018;Wechsler, 1987), but to minimize practice effects, we introduced four alternative stories from the Rivermead Behavioral Memory Test (Kazui et al., 2003). We administered the Trail Making Test (TMT) to evaluate executive function. To control for the effects of age-related motor function impairments, we used the TMT delta, calculated as the time to complete Part B minus Part A.

### 2.3 Image acquisition

Whole-brain imaging was performed on a 3T Siemens Magnetom Verio MRI scanner (Siemens, Erlangen, Germany). Functional images were acquired with a T2*-weighted echo-planar imaging (EPI) sequence, which included 39 axial slices with a thickness of 3.5 mm and an in-plane resolution of 3.5 × 3.5 mm. The repetition time (TR) was 2000 ms, echo time (TE) was 25 ms, the flip angle was 75°, the field of view (FOV) was 224 × 224 mm, and the matrix was 64 × 64. The first five volumes were discarded to allow for T1 equilibration effects. The block-design fMRI used n-back tasks with face and digit stimuli. Following the functional scan combined with WM tasks, high-resolution structural brain images were collected using a T1-weighted, 3D magnetization prepared rapid acquisition gradient echo (MP-RAGE) pulse sequence with a voxel size of 1 × 1 × 1 mm^3^, 208 axial slices, an FOV of 256 × 256 mm, and a matrix = 256 × 256.

### 2.4 Visual WM task: face n-back task

The face n-back task, conducted as the visual WM task at baseline, was administered in this follow-up test to observe the longitudinal changes. It was a block-design task consisted of 0-back, 1-back, and rest conditions. Each condition included 4 blocks, each lasting for 32 seconds, preceded by a 4-second instruction period. The face stimuli consisted of neutral faces of Japanese university students (26 females and 26 males) and were presented sequentially, with each stimulus appearing on the screen for 2000 ms, followed by a stimulus onset asynchrony (SOA) of 4000 ms. Participants responded using their dominant (right) hand. In the 0-back task, participants were instructed to press the left button with their index finger when the displayed face stimuli disappeared. For the 1-back task, participants were to press the left button with their index finger if the current face stimuli matched the one presented immediately before; otherwise, they were to press the right button with their middle finger. Of the 32 trials (8 trials × 4 blocks) for 1-back task, 12 required a “match” response (left button) and 20 required a “non-match” response (right button). During the rest condition, participants focused on a fixation cross in the middle of the screen. Further details are provided inGuo et al. (2021).

The task load, that is, 1-back for the face stimuli, was designed to ensure relatively high behavioral accuracy during fMRI scanning, allowing for meaningful and valid interpretations of neural efficiency (i.e., decreased neural activity with comparable behavioral performance) within the block-design fMRI experiment. Our previous studies indicated that a 2-back task is too demanding for older adults (accuracy below 45%) (Kawagoe & Sekiyama, 2014) while the 1-back task successfully detected improved neural efficiency induced by a multimodal intervention (Nishiguchi et al., 2015). Based on these findings, we used the 0-back and 1-back tasks to assess the short-term intervention effect (Guo et al., 2021) and repeated the task in this study to track the long-term changes in neural efficiency.

### 2.5 Verbal WM task: digit n-back task

Previous studies have indicated that the effect on verbal WM is a key cognitive benefit induced by musical instrument training (Vetere et al., 2024;Wang et al., 2023). To further investigate the neural mechanisms underlying this musical instrument training-related benefit in verbal WM processing, we introduced a digit n-back task in the follow-up assessment. Our previous study indicated that for digit stimuli, a 1-back task resulted in a ceiling effect with accuracy exceeding 90%, while a 2-back task was too challenging for older adults, with accuracy falling below 60% (Kawagoe & Sekiyama, 2014). Therefore, to ensure performance levels without introducing excessive difficulty—since relatively high accuracy is crucial for acquiring meaningful data—this study employed a 1.5-back task for the digit stimuli, asking whether the current item matches the item from one or two trials earlier. The verbal WM task consisted of 12 blocks, with 4 blocks each for 0-back, 1.5-back, and rest condition. Each block included nine trials, lasting 36 seconds, preceded by a 4-second instruction period. AsFigure 2shows, in the 0-back and 1.5-back conditions, the digit stimuli were integers ranging from 0 to 9. The stimuli were displayed sequentially, each stimulus for 2000 ms, with an SOA of 4000 ms. A black central fixation cross (+) was displayed between the stimuli. Responses not made before the next stimulus were recorded as misses. Prior to scanning, participants practiced outside the scanner with different stimulus sequences. For the 0-back task, participants pressed the left button with the index finger of the right hand as soon as the digit stimulus disappeared. Of the 36 trials (9 trials × 4 blocks) for 1.5-back task, 16 required a “match” response (left button) and 20 required a “non-match” response (right button). Participants were instructed to give correct response, that is, hit for the same and correct rejection for the different trials, as quickly as possible. During the rest condition, participants were instructed to focus on a fixation cross in the center of the screen.

**Fig. 2.**
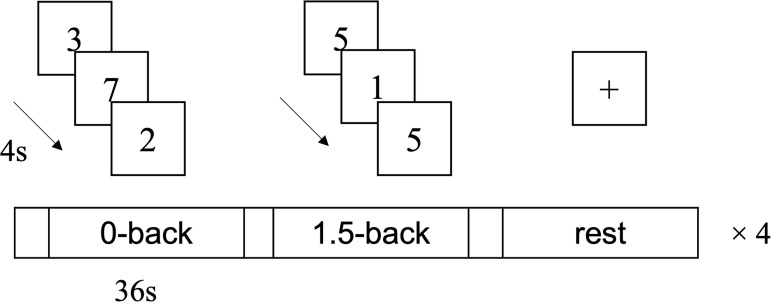
The block-design verbal working memory task comprised 12 blocks, with 4 blocks each for the 0-back, 1.5-back, and rest conditions. Each block began with a 1-second instruction period, followed by nine trials lasting a total of 36 seconds. In the 0-back task, participants were instructed to press the button when the displayed digit stimuli disappeared. For the 1.5-back task, participants were asked to determine whether the displayed digit matched the digit from one or two trials earlier. In the rest condition, they were instructed to keep their focus on a fixation cross in the center of the screen.

### 2.6 Structural MRI data processing

Brain volumes were measured using FreeSurfer version 7.1.1 (Fischl, 2012;Reuter et al., 2012). Image processing included the following procedures: motion correction, skull stripping, Talairach transformation, segmentation of the subcortical white matter (WM) and deep gray matter (GM) structures, intensity normalization, tessellation of the GM/WM boundary, surface deformation along intensity gradients for optimal placement of GM/WM and GM/cerebrospinal fluid borders, and cortical parcellation with submillimeter precision into units based on gyral and sulcal structure. Manual reviews and edits were performed on segmentations to correct geometric inaccuracies. Bilateral volumes of the caudate, putamen, and cerebellum cortex were extracted. Total intracranial volume (TIV) was also estimated for each image. Striatal and cerebellar volumes were normalized to percent volume (%) relative to baseline TIV to account for individual head size variations (Whitwell et al., 2001).

### 2.7 Task-related fMRI data processing

We preprocessed the fMRI data using SPM 12 (Wellcome Department of Cognitive Neurology, London) and MATLAB R2021a (The Mathworks Inc., United States) with the following steps: First, we set the origin of the EPI to the anterior-posterior commissure for each participant with manual review and adjustment. Second, slice timing correction was applied to adjust for acquisition time differences. Third, we corrected for head motion by realigning the time series images to the first functional image. Fourth, the realigned functional images were coregistrated to the T1 structural image and then normalized to a Montreal Neurological Institute (MNI) space. Subsequently, the normalized images were finally smoothed using an 8-mm FWHM (full width at half maximum) Gaussian filter. A high-pass filter (1/128 Hz) was used to remove low-frequency noise, and an autoregressive model was employed to correct for temporal autocorrelations.

Activated voxels in each condition (0-back, 1-back, and rest for the face tasks and 0-back, 1.5-back, and rest in the digit task) were modeled using a statistical model containing a boxcar function convolved with a canonical hemodynamic response function. Linear contrasts provided participant-specific estimates for each effect. For the face task, brain activation associated with visual WM was identified by contrasting the 1- versus 0-back conditions. For the digit task, activation related to verbal WM was analyzed by contrasting the 1.5- versus 0-back conditions. These estimates were then used in an SPM12-based whole brain analysis with participant as a random effect.

### 2.8 Functional connectivity data preprocessing

We used the CONN toolbox (https://www.nitrc.org/projects/conn) to analyze the FC during the face and digit tasks. Data preprocessing within CONN involved addressing the potential confounding effects from head motion artifacts, cerebrospinal fluid, and blood oxygen level-dependent (BOLD) signals, using default settings. The data were bandpass filtered between 0.008 and 0.09 Hz. Seed-to-voxel analyses were performed by correlating the mean time series from the selected seeds with those from other voxels. The seeds were selected to test our hypothesis that the functional preservation of the striatum and cerebellum mediates the beneficial musical instrument training effects on WM processing. The seeds were defined using the FSL Harvard–Oxford maximum likelihood subcortical atlas within the CONN toolbox, including the left and right putamen, caudate, and cerebellar regions involved in WM or language processing (lobule VI, VIIa Crus I, VIIa Crus II, lobule VIIb, and lobule IX) (Guell et al., 2018;Habas et al., 2009;Küper et al., 2016;Stoodley & Schmahmann, 2009;Šveljo et al., 2014;Viñas-Guasch et al., 2022). The cluster sizes of each seed region are provided inSupplementary Table S1. In addition to the hypothesis-driven seed selection, a result-based approach was also applied. Clusters were defined as regions of interest and selected as seeds if they (1) were located outside the striatum and cerebellum (areas already defined as seeds based on the hypothesis) and (2) met one of the following criteria: (a) revealed a significant group-by-time interaction in the longitudinal activation data for the face task or (b) showed a significant group difference in the cross-sectional activation data for the digit test. The statistical thresholds were set at*p*< 0.001 uncorrected for multiple comparisons at the voxel level, and*p*< 0.05 family-wise error (FWE)-corrected for multiple comparisons at the cluster level. First-level analysis involved computing Fisher-transformed connectivity values. The mean task-related BOLD time course of each seed was correlated with that of each voxel using a general linear model and bivariate correlation analysis weighted for the hemodynamic response function. Individual correlation maps were generated by the correlation coefficients. Second level analysis for the face task involved an ANOVA with time of assessment (baseline vs. follow-up) and condition (0-back vs. 1-back) as the within-subject variables, and group (continue vs. stop) as the between-subject variable, to assess the musical instrument training effects across 4 years. For the digit task, a two-way ANOVA was conducted to examine group differences in the FC during verbal WM processing, with condition (1.5-back vs. 0-back) as the within-subject variable and group (continue vs. stop) as the between-subject variables. Statistical thresholds were set at*p*< 0.001 uncorrected for multiple comparisons at the voxel level, and*p*< 0.05, FWE corrected for multiple comparisons at the cluster level.

### 2.9 Statistical analysis

We conducted a two-way ANOVA to assess the longitudinal changes in behavior, striatal and cerebellar GMV, and the brain activation during face task among participants who continued melodica training versus those who quit but engaged in other activities. The ANOVA used group as the between-subject variable and time as the within-subject variable. When the “group × time” interaction was significant, we further investigated the simple main effect of time to observe changes in each group. Intervention effects were deemed significant if the “group × time” interaction and the simple main effect of time in any one group were both significant. To identify cross-sectional group differences in brain activation for the digit task between the continue group and the stop group, we performed a one-way ANOVA with group as the between-subject variables. The statistical thresholds for task-related activation analysis were set at*p*< 0.001 uncorrected for multiple comparisons at the voxel level, and*p*< 0.05 FWE-corrected for multiple comparisons at the cluster level.

Additionally, Spearman’s partial correlation was conducted to evaluate the association between structural and functional results and behavioral changes. We focused on the cognitive measurements showing significant intervention effects, specifically the composite score of WM. Structural changes were assessed in regions where the musical instrument training effects were revealed. Parameter estimates of BOLD values were extracted using MarsBaR software (Brett et al., 2002) from clusters where group-by-time interaction or group differences were identified. Individual-level FC values were extracted using the CONN toolbox. Correlations were assessed among changes in behavior, structure, and function, controlling for age. We analyzed all the behavioral and structural changes, extracted BOLD values, and FC values in R (R Core Team, 2023), with the significance level set at*p*< 0.05. Outliers were removed if they exceeded 3 standard deviations.

## 3 Results

### 3.1 Characteristics of participants

There were no statistically significant group differences in age, years of education, MMSE, sex ratio, or the original group membership during intervention, asTable 2shows. In comparison of the leisure participation and social engagement, asTable 3shows, the two groups showed no statistically significant difference in engagement in physical, cognitive, or social activities.

**Table 2.** Characteristics of participants.

	Continue group		Stop group		
	Mean	*(SD)*	Mean	*(SD)*	*p* -value
Age (years)	77.85	*(4.30)*	76.00	*(5.44)*	0.314
Academic years	13.69	*(1.97)*	13.63	*(2.09)*	0.935
MMSE	28.62	*(1.04)*	28.74	*(1.10)*	0.756
Sex (female/male)	10/3		13/6		0.599
Group during intervention (Intervention/ Waiting group)	5/8		11/8		0.280

**Table 3.** Leisure participation and social engagement in two groups.

	Continue group		Stop group		
	Mean	*(SD)*	Mean	*(SD)*	*p* -value
Physical exercise	7.23	*(10.44)*	13.66	*(12.77)*	0.144
Cognitive activity	6.08	*(2.06)*	6.37	*(10.25)*	0.905
Social engagement	1.31	*(0.48)*	1.32	*(0.58)*	0.966

Physical exercise and cognitive activity are indicated by times per month. For social engagement, points represent sum of three items (yes = 1, no = 0): (1) participation in a paid job, (2) participation in volunteer activity, and (3) not frequently being alone during the daytime.

### 3.2 Behavioral results

The t-test detected no significant group difference at baseline in any of the three measures of verbal WM (*p*= 0.358, Cohen’s*d*= -0.345), delayed story recall (*p*= 0.091, Cohen’s*d*= -0.070), or TMT delta (*p*= 0.787, Cohen’s*d*= -0.090). The “group × time” ANOVA revealed a significant interaction in verbal WM [*F*(1, 30) = 5.095,*p*= 0.031, generalized η^2^= 0.038] and TMT delta [*F*(1, 30) = 5.980,*p*= 0.021, generalized η^2^= 0.044], but not in delayed story recall [*F*(1, 30) = 0.536,*p*= 0.470, generalized η^2^= 0.004]. The “group × time” interactions in verbal WM and TMT delta remained significant after FDR correction (verbal WM: corrected*p*= 0.047, TMT delta: corrected*p*= 0.047, delayed story recall: corrected*p*= 0.470).

**Fig. 3.**
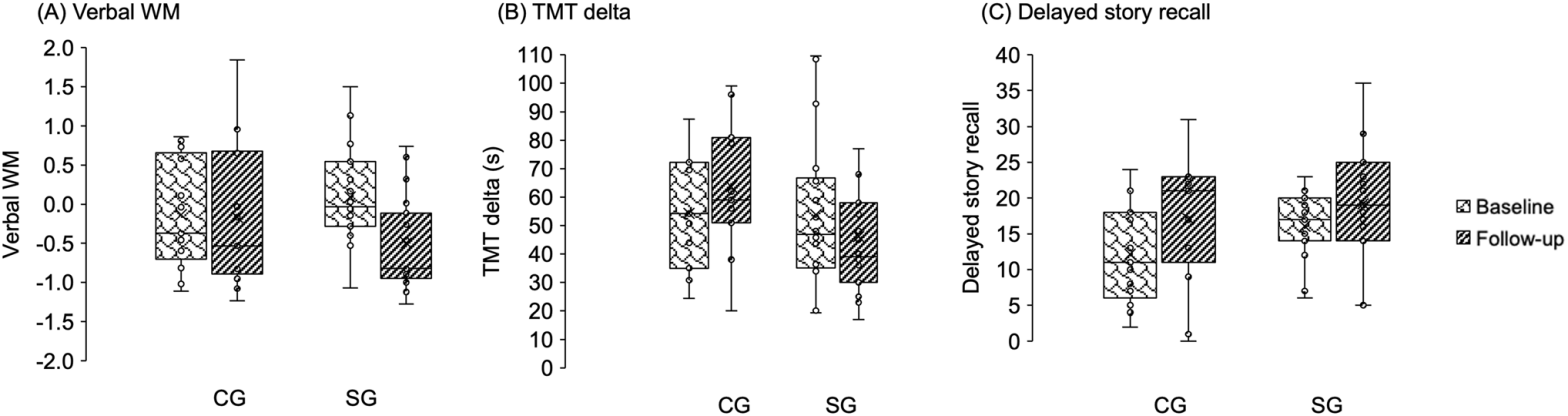
Behavioral performance in baseline and follow-up tests in the two groups. (A) Verbal working memory measure, the composite score of Digit Span (Forward and Backward) and Verbal Fluency (Semantic and Phonological). (B) Trail making test delta (time to complete Part B minus Part A). (C) Delayed story recall. The box represents the interquartile range (IQR), extending from the first quartile (Q1) to the third quartile (Q3), with the horizontal black line inside each box indicating the median score. The error bars (whiskers) extend from the box to the minimum and maximum values within 1.5 times the IQR. WM, working memory; TMT, Trail Making Test; CG, continue group; SG, stop group.

Figure 3shows participants’ performance on verbal WM, TMT delta, and delayed story recall. For verbal WM, the simple main effect of time was only significant in the stop group [*F*(1, 18) = 10.695,*p*= 0.004, generalized η^2^= 0.197], decreasing from 0.09 to -0.5. The continue group showed no significant simple main effect of time in WM [*F*(1, 12) = 0.069,*p*= 0.798, generalized η^2^= 0.001]. The simple main effect of time was significant in neither group on TMT delta [continue group:*F*(1, 12) = 4.026,*p*= 0.068, generalized η^2^= 0.251; stop group:*F*(1, 18) = 0.681,*p*= 0.420, generalized η^2^= 0.036].

### 3.3 Effects on GMV preservation and correlation with verbal working memory

Two groups did not differ in striatal and cerebellar volumes at baseline (right hemisphere: caudate:*p*= 0.145, putamen:*p*= 0.311, cerebellum:*p*= 0.539; left hemisphere: caudate:*p*= 0.121, putamen:*p*= 0.972, cerebellum:*p*= 0.575). AsFigure 4shows, the “group × time” ANOVA revealed a significant interaction on the volume of the right putamen [*F*(1, 30) = 8.049,*p*= 0.008, generalized η^2^= 0.017] and the right cerebellum [*F*(1, 30) = 4.413,*p*= 0.044, generalized η^2^= 0.011]. The “group × time” interaction remained significant on the volume of right putamen (corrected*p*= 0.048) but not on the right cerebellar volume (corrected*p*= 0.132) after FDR correction. The “group × time” interaction was not significant in the GMV of the striatum (putamen or caudate) or the cerebellum in the left hemisphere or the right caudate. For the GMV in right putamen, only the stop group showed significant decrease [*F*(1, 18) = 19.656,*p*< 0.001, partial η^2^= 0.038], while the continue group showed no significant simple main effect of time [*F*(1, 12) = 0.844,*p*= 0.376, partial η^2^= 0.007]. Both groups showed significant decline in the right cerebellar volume [continue group: [*F*(1, 12) = 16.800,*p*= 0.002, partial η^2^= 0.066; stop group [*F*(1, 18) = 47.831,*p*< 0.001, partial η^2^= 0.198].

**Fig. 4.**
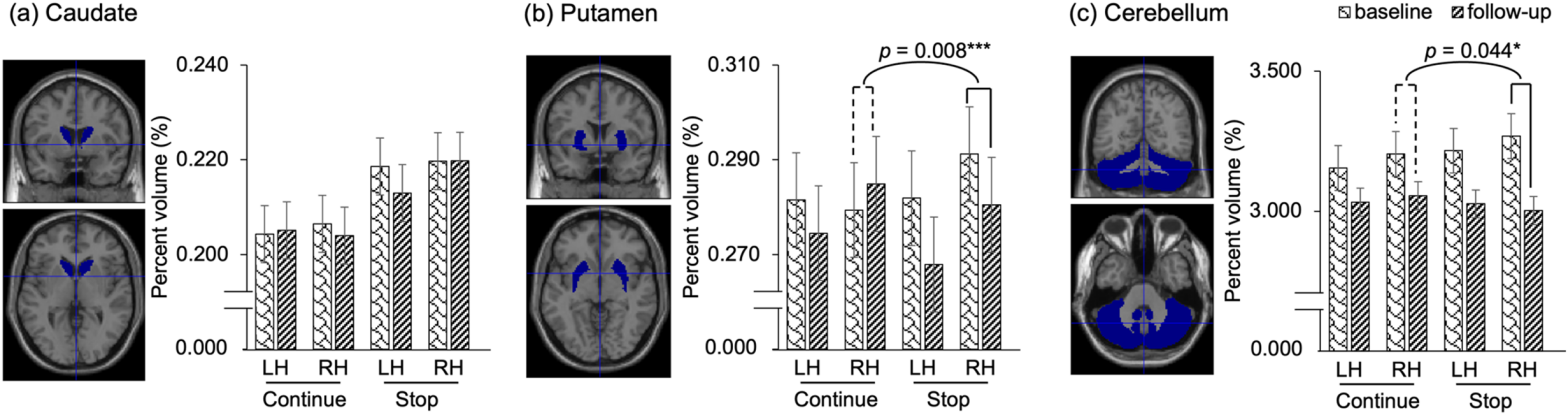
Gray matter volume changes in the continue group (n = 13) and stop group (n = 19): (a) Caudate GMV. (b) Putamen GMV. (c) Cerebellum GMV. Percentage volume (%) was calculated by dividing each volume by the TIV for each participant. Error bars represent the standard error of mean. LH, left hemisphere; RH, right hemisphere. *,*p*< 0.05; ***,*p*< 0.01.

**Fig. 5.**
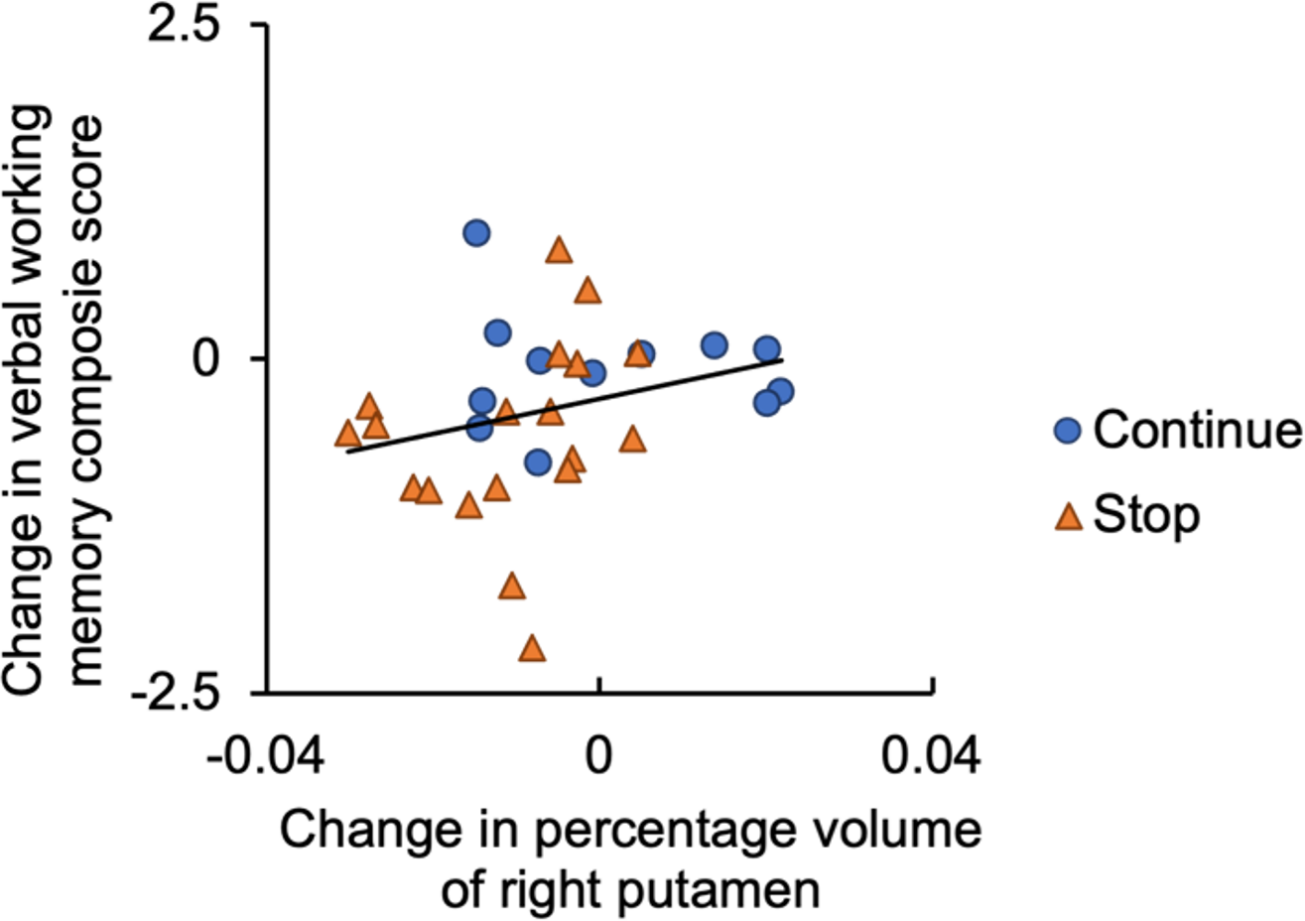
Correlation between changes in the right putamen volume and the working memory composite score (rho = 0.421,*p*= 0.016).

Figure 5shows Spearman’s correlation between the changes in the composite score of WM and the GMV change in the right putamen where significant musical instrument training effects were revealed. Better preservation in behavioral performance on WM tasks significantly correlated with better preservation of the right putamen GMV (rho = 0.421,*p*= 0.016), with one GMV data point excluded for exceeding the mean by more than 3 SD. The correlation remained significant after FDR correction (corrected*p*= 0.025).

### 3.4 Longitudinal change in visual WM processing: the face n-back task

#### 3.4.1 Behavioral performance and brain activation during the face n-back task

Responses were 88.58% correct in the face 1-back task (SD = 15.84%) and 97.03% correct in the 0-back task (SD = 7.18%). The whole-brain analysis detected no group-by-time interaction on brain activation during the face n-back task.

#### 3.4.2 Functional connectivity during the face n-back task

We analyzed the FC using subregions of the striatum and cerebellum as seeds based on the hypothesis. AsTable 4shows, significant group × time × condition interaction was revealed when choosing the right cerebellum IX and the right cerebellum Crus I as seeds. No significant results detected using the putamen, caudate, or other subregions of the cerebellum as seed which were defined based on hypothesis. No seeds were defined based on the activation results, as the whole-brain analysis revealed no significant clusters.

**Table 4.** Regions where the functional connectivity showed training effect in the follow-up study (cluster-level*p*< 0.05, FWE corrected).

	MNI coordinates				
Region	x	y	z	*p* -value (FWE correction)	Cluster size
Seed: Right cerebellum IX					
Right supramarginal gyrus	52	-34	42	0.002	179
Seed: Right cerebellum Crus I					
Right cerebellum IX to VIII	16	-50	-50	0.041	96

FWE, family-wise error; MNI, Montreal Neurological Institute.

As shown inFigure 6, the FC between the right cerebellum IX seed and the right supramarginal gyrus (rCerebIX-rSMG FC) and the FC between the right cerebellum I seed and the right cerebellum IX to VIII showed significant group × time ×condition interaction. In the 1-back over 0-back condition, the continue group showed decrease while the stop group showed increase in the rCerebIX-rSMG FC; the continue group showed increase while the stop group showed decrease in the rCerebI-rCereb VIII to IX FC. Both the decrease in rCerebIX-rSMG FC and the increase in the rCerebI-rCereb VII to IX FC marginally correlated with better preserved verbal WM composite score (rCerebIX-rSMG FC: partial ρ = -0.381,*p*= 0.032, FDR corrected*p*= 0.051; rCerebI-rCereb VII to IX FC: partial ρ = 0.352,*p*= 0.048, FDR corrected*p*= 0.051).

**Fig. 6.**
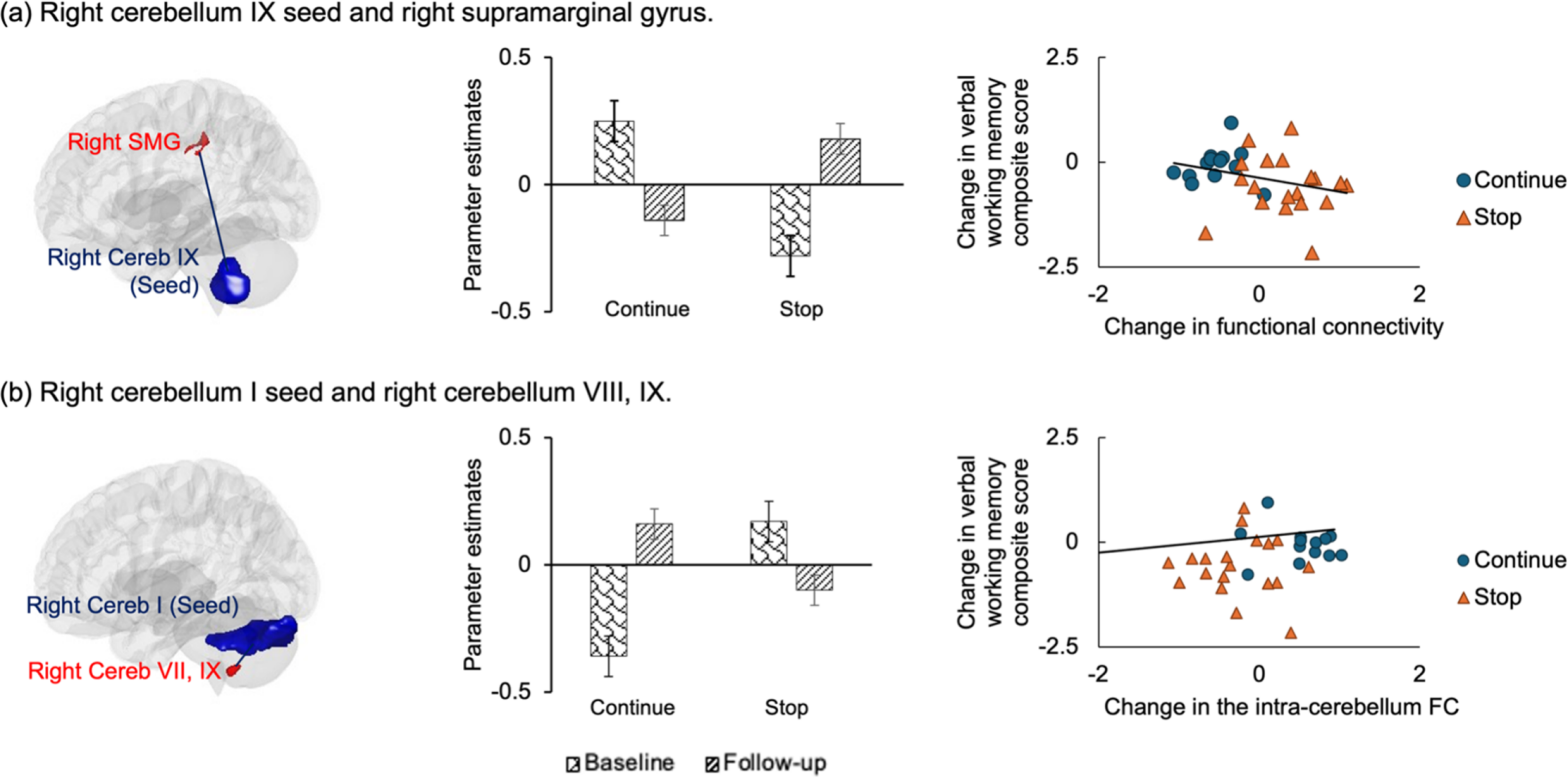
The training effects were revealed by significant group × time × condition on (a) the FC between the right cerebellum IX seed and the right supramarginal gyrus, and (b) the FC between the right cerebellum I seed and the right cerebellum VIII and IX (Cluster level FWE corrected*p*< 0.05). Error bars represent standard deviation (SD). Both FC significantly correlated with the behavioral performance change in the verbal WM: rCerebIX-rSMG, partial ρ = -0.381,*p*= 0.032, FDR corrected*p*= 0.051; rCerebI-rCerebVIII~IX, partial ρ = 0.352,*p*= 0.048, FDR corrected*p*= 0.051. FC, functional connectivity; rCereb, right cerebellum; rSMG, right supramarginal gyrus.

### 3.5 Cross-sectional comparison in verbal WM processing: the digit n-back task

#### 3.5.1 Behavioral performance and brain activation during the digit n-back task

Responses were 85.65% correct in the digit 1.5-back task (SD = 13.23%). The whole-brain analysis computed contrast images for 1.5-back versus 0-back to assess the brain activity during the verbal WM task in both groups. Between-group differences are shown inTable 5andFigure 7a. The continue group showed greater activation in bilateral cerebellum than the stop group. Subsequently, we extracted parameter estimates in the cluster and investigated the associations between the activation and change in verbal WM assessed by offline Digit Span and Verbal Fluency tasks. AsFigure 7bshows, age-controlled Spearman’s correlation revealed a marginally significant correlation between greater activation and better preserved WM performance (partial ρ = 0.347,*p*= 0.051). The correlation remained marginally significant after FDR correction (corrected*p*= 0.051).

**Fig. 7.**
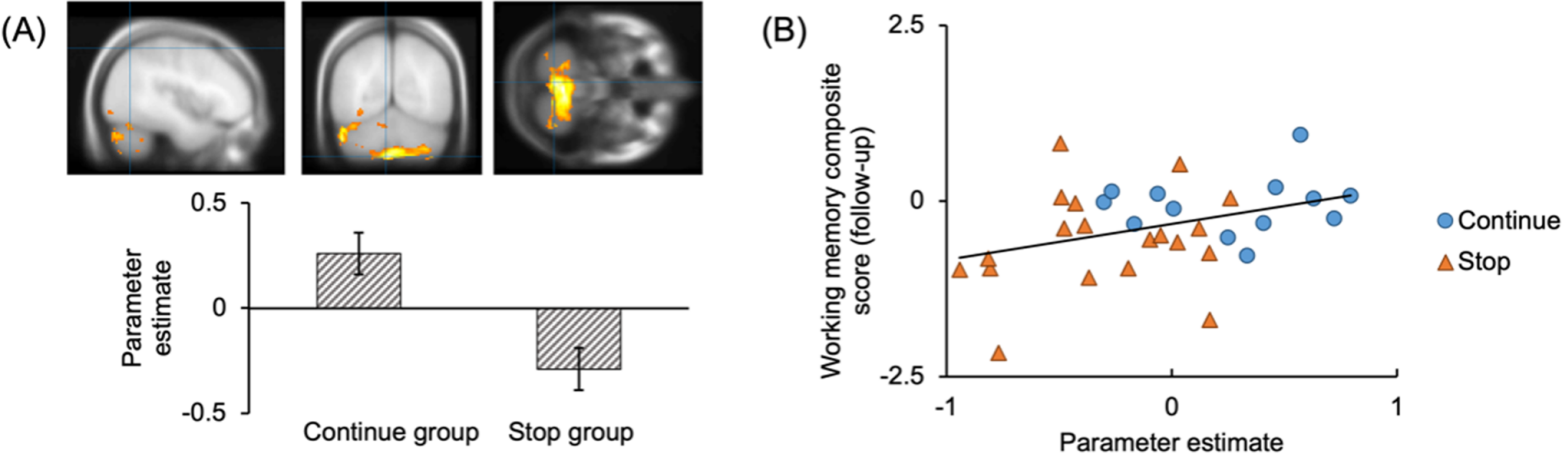
Contrast image for number 1.5-back versus 0-back showed a significant difference between two groups in a cerebellum cluster encompassing both hemispheres, which includes VI, Crus II, VII b, VIII, IX (Cluster level FWE corrected*p*< 0.05). Error bars represent standard deviation (SD). (A) Brain activation (BOLD signals) for the 1.5-back over 0-back condition. (B) Correlation between the BOLD signals and the behavioral verbal working memory performance. Age-controlled partial correlation between the cerebellar activation and the working memory composite score: rho = 0.348,*p*= 0.051.

**Table 5.** Brain regions in which contrast images for 1.5-back versus 0-back showed greater activation in the continue group than the stop group (cluster level*p*< 0.05, FWE corrected).

	MNI coordinates				
Region	x	y	z	t-value	Cluster size
Cerebellum	-6	-64	-54	4.24	117

FWE, family-wise error; MNI, Montreal Neurological Institute.

#### 3.5.2 Functional connectivity during the digit n-back task

AsTable 6shows, among the seeds selected based on the hypothesis, group difference was revealed significant in the FC between the left cerebellum IX seed and the brainstem, specifically in the pons. AsFigure 8shows, compared with the stop group, the continue group showed a weaker cerebellar–brainstem FC, which significantly correlated with the working memory change (partial rho: -0.56,*p*< 0.01). The correlation remained significant after FDR correction (corrected*p*= 0.005). No seeds were defined based on the activation results, as the clusters showing a significant group difference in the whole-brain analysis were located in the cerebellum, which had already been defined as seeds based on the hypothesis.

**Table 6.** The regions which showed stronger functional connectivity in the continue group than the stop group in the 1.5-back over 0-back condition (cluster-level*p*< 0.05, FWE corrected).

	MNI coordinates				
Region	X	y	z	*p* -value (FWE correction)	Cluster size
Seed: Left cerebellum IX					
Brainstem	-2	-20	-36	<0.001	234

FWE, family-wise error; MNI, Montreal Neurological Institute.

**Fig. 8.**
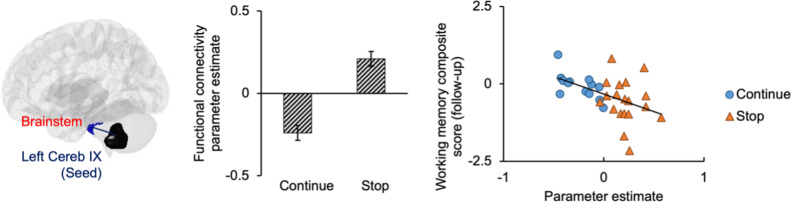
The group difference was revealed on the left Cereb IX–brainstem functional connectivity in the 1.5-back versus 0-back condition. Cluster level FWE corrected*p*< 0.05. Error bars represent standard deviation (SD). The functional connectivity was significantly correlated with the working memory change: partial rho = -0.57,*p*< 0.01, FDR corrected*p*= 0.005. Cereb, cerebellum.

## 4 Discussion

This study investigated the musical instrument training effects on age-related cognitive decline by monitoring behavioral and structural changes across 4 years in healthy older adults. Participants who consistently engaged in musical instrument training showed less decline in verbal WM and better preservation of GMV in the right putamen compared with those who discontinued training. Moreover, better preserved performance on behavioral verbal WM tasks was correlated with better preserved GMV in the right putamen. Regarding brain activation and FC during WM tasks, the continue group showed a lower cerebellum–pons FC during verbal WM processing, which was significantly correlated with better preserved behavioral performance on offline verbal WM tasks. The lower FC and its correlation with improved verbal WM performance suggest that musical training enhances neural efficiency. Additionally, greater cerebellar activation during the verbal WM task indicated that these effects might be due to the cerebellum’s enhanced ability to recruit intra-cerebellar neural resources, reducing reliance on the overactivation of other brain areas to meet the gap between neural resources and cognitive demands. The increased intra-cerebellar FC and decreased cerebellar–cortical (rCerebIX-rSMG) FC during the face task consistently supported the role of musical training in enhancing the cerebellum’s capacity. Thus, this study provides combined evidence for the musical instrument training effects on cerebellar function. Together with the findings on putamen volume, the results emphasize the role of subcortical neuroplasticity in the cognitive benefits induced by musical training.

This long-term follow-up study addressed the limitations of previous cross-sectional comparisons between older musicians and age-matched non-musicians. First, it provided longitudinal evidence of the causal musical instrument training effects on cognitive and brain reserve in healthy older adults. Second, the two demographically homogeneous groups showed no significant behavioral or structural differences at baseline, with all participants musically untrained, thereby ensuring effective control of potential confounding factors. Moreover, the extended training period limited the potentially confounding effect of environmental novelty which might influence the short-term intervention effects in musically untrained participants. Furthermore, the long test interval in this study facilitated a deeper investigation into the musical instrument training effects on cognitive aging (Rogers & Metzler-Baddeley, 2024). To the best of our knowledge, this is the first longitudinal study to demonstrate the benefits of initiating musical instrument training in older age for mitigating age-related memory decline, brain shrinkage, and dysfunction in healthy older adults over several years.

### 4.1 The musical instrument training effects on behavioral performance

Participants who consistently engaged in musical instrument training showed less decline in verbal WM across 4 years, demonstrating the musical instrument training effects in mitigating age-related cognitive decline. Consistent with previous cognitive aging studies, the stop group showed significant verbal WM decline across 4 years in their older age (Hultsch et al., 1992;Nyberg et al., 2012;Park et al., 2002). The well-preserved verbal WM in the continue group was consistent with less WM decline in older musicians compared with age-matched non-musicians (Böttcher et al., 2022;Fauvel et al., 2014;Gray & Gow, 2020;Hanna-Pladdy & Gajewski, 2012;Mansens et al., 2018;Vetere et al., 2024;Yamashita et al., 2022), and specified the musical instrument training effects experience initiated in older age. This also aligned with the effects of our previous 10-week musical instrument training intervention on improving the verbal WM (Wang et al., 2023). In addition to the short-term intervention identifying improved verbal WM as an early and essential cognitive benefit induced by musical instrument training, this study further indicated the effects of extended training on mitigating age-related decline on verbal WM. Additionally, this study addressed the limitations of previous research regarding study design. Earlier studies were weakened by the absence of an active control, a limitation this study overcame by including a stop group who remained actively engaged in group leisure activities at the same welfare center. Furthermore, unlike short-term interventions involving musically untrained healthy older adults, where the observed benefits might stem from the novelty of engaging in a new leisure activity, participants in this study underwent 4 years of continuous musical instrument training. This prolonged exposure allowed them to become familiar with the training, making it more likely that the observed effects can be attributed to the training itself rather than novelty.

However, this study did not replicate the musical instrument training effects on verbal memory (delayed story recall) observed in our previous 16-week intervention with the same participants but a larger group size (Guo et al., 2021). Both the continue group and the stop group showed improvement in the follow-up assessment.

The improvement observed in the follow-up assessment may reflect the melodica training effects on verbal memory, which might have persisted for more than 3 years after the conclusion of the 12- to 16-week program (Guo et al., 2021). Alternatively, the improvement could also suggest a training effect due to repeated participation in the tests. Although we used alternative stories to minimize practice effects, learning memory strategies through repeated test participation may have also contributed to improved performance (Gavett et al., 2016;Lo et al., 2012;Theisen et al., 1998).

Regarding the TMT delta, though the group-by-time interaction was significant, neither group showed significant simple main effect of time. The improvement on TMT delta was only observed in the 6-month individualized piano intervention among healthy older adults (Bugos et al., 2007), not in any of our previous group melodica interventions (Guo et al., 2021;Wang et al., 2023). The difference in the type of musical instrument used might explain the inconsistency. Bimanual coordination may play a role in the musical instrument training effects on executive control (Bugos, 2019).

### 4.2 Effects on the preservation of brain structure: the neuroplasticity of putamen

The continue group preserved the GMV of the right putamen across 4 years, whereas the stop group showed a significant decrease. The better preserved putamen GMV in the continue group aligns with previous findings showing an association between musical instrument training and greater putamen GMV previously noted in young musicians (Vaquero et al., 2016).

Moreover, the well-preserved GMV was correlated with less decline in verbal WM, suggesting the preservation of the putamen mediated the effects on verbal WM. The putamen interconnects the motor areas (Di Martino et al., 2008;Lehericy, 2004), and convergence of neuroimaging data indicated that the motor system supports WM processing (Marvel et al., 2019). The involvement of the putamen in learning (Brovelli et al., 2011;Haruno & Kawato, 2006;Zhao et al., 2021) and WM processing (Chang et al., 2007;Dodds et al., 2009;Moore et al., 2013) is well documented, with the striatum demonstrated to play a critical role in learning and the transfer of effects to executive component of WM (Dahlin et al., 2009;Kühn et al., 2013). Our previous 16-week melodica intervention in healthy older adults (a) showed a decreased FC between the left putamen and the right superior temporal gyrus during the visual WM processing after training and (b) associated the decreased FC with improved verbal memory (Guo et al., 2021). A 4-week cello training also showed effects on increased activation in the right putamen during listening to trained sequences in young adults (Wollman et al., 2018). Combining evidence on the GMV, FC, and activation, these studies emphasize neuroplasticity of the putamen induced by musical instrument training. This neuroplasticity of the putamen may act as a mediator of the effects of musical training on WM processing.

The lateralized effect observed in our study, preservation of the right but not the left putamen, may be explained by the functional specialization of the two hemispheres. While both the left and right putamen contribute to cognitive and motor functions, previous research has shown that they specialize in distinct processes. The left putamen is more directly involved in language comprehension and production, often coactivating with regions that support these functions (Viñas-Guasch & Wu, 2017). In contrast, the right putamen is more engaged in visual and orthographic processing, as well as motor task execution, which are crucial components of musical instrument training. This specialization in visual WM and motor function likely explains why the right putamen, rather than the left, was more strongly involved in our study. Furthermore, structural differences within the right putamen have been linked to proprioceptive sensibility and motor functions (Goble et al., 2012). Given that musical instrument training involves integrating visual information with motor actions, such as reading musical notation and translating it into motor sequences with or without visual aid, the right putamen’s role in these processes may have been strengthened. This could account for the observed preservation of GMV in the right putamen, particularly in participants who continued the training.

### 4.3 Effects on enhanced cerebellar function in WM processing

This study provided combined evidence supporting the enhanced ability in the cerebellum to recruit intra-cerebellar neural resources in response to increasing WM demands. First, the verbal WM task revealed lower FC between the cerebellum and pons in the continue group. The lower cerebellum–pons FC was significantly correlated with better preserved verbal WM performance, as indicated by the composite score of Digit Span and Verbal Fluency. These findings suggest higher neural efficiency in WM processing related to musical training. Combined with other results, the decreased cerebellar–pons FC seems to be a part of the cerebellum’s enhanced ability to recruit intra-cerebellar resources. First, the continue group showed greater activation in the bilateral cerebellum during the digit task. With increasing WM demand, young adults showed increased activation in cerebellum as a typical effect of cognitive load (Desmond et al., 1997;Küper et al., 2016;Peterburs et al., 2019). In contrast, in addition to cerebellar activation, healthy older adults engage the fronto-parietal network during successful WM processing, especially under higher executive demands (Luis et al., 2015). Therefore, a well-activated cerebellum, with less reliance on overactivation of other brain areas for compensation, may reflect a younger activation pattern in response to cognitive demands.

Second, the longitudinal changes in FC during visual WM task provided additional support for the enhanced cerebellar function and the potentially improved neural efficiency. The continue group showed an increased intra-cerebellar FC and a decreased cerebellar–cortical (rCerebIX-rSMG) FC over the 4 years, in contrast to the changes observed in the stop group. Consistent with the cross-sectional results from the verbal WM task, the increased intra-cerebellar FC supported the cerebellum’s enhanced ability to recruit intra-cerebellar neural resources, while the decreased rCerebIX-rSMG FC reflected a reduced reliance on cortical overactivation to bridge the “supply–demand gap”—an insufficiency or gap between available neural resources and task demands (Cabeza et al., 2018). Several studies have shown that in healthy aging, cortical areas overactivate to compensate for the subcortical dysfunction. A cross-sectional study found that, in older adults, behavioral mobility was positively correlated with subcortical activation and negatively correlated with prefrontal activation, suggesting the frontal overactivation compensates for subcortical dysfunction (Kawagoe et al., 2015). Longitudinal studies in WM training have also demonstrated changes in the underlying skills following training (Dahlin et al., 2009), and the demonstrated positive behavioral changes were accompanied by a decrease in fronto-parietal activity (Chein & Schneider, 2005;Kelly & Garavan, 2005;Kelly et al., 2006) and an increase in subcortical regions (Dahlin et al., 2008;Olesen et al., 2004;Tomasi et al., 2004). Physical and cognitive exercise interventions have also shown decreased PFC activation during WM processing in older adults (Nishiguchi et al., 2015). Lower PFC activation and greater subcortical activation in WM processing were reported in young musicians and associated with better performance (Alain et al., 2018;Pallesen et al., 2010). Our previous intervention, conducted in a larger cohort from the same participant group, demonstrated decreased FC between the left putamen and the right superior temporal gyrus during visual WM processing after training. This decrease was correlated with improved verbal memory, consistently linking enhanced behavioral performance with reduced cortical–subcortical FC (Guo et al., 2021).

Taken together, this study provided combined evidence for better preserved cerebellar function in the continue group. The findings highlighted the cerebellum’s enhanced ability to recruit intra-cerebellar neural resources to perform the demanding WM task, thereby reducing the need for the overactivation of cortical areas and the brainstem to bridge the “supply–demand gap.” The enhanced cerebellar function may be attributed to the nature of melodica playing, which requires precise finger control to press the keys and effective breath control to produce sound. Coordinating these elements seamlessly relies on the cerebellum, which plays a central role in integrating and coordinating complex motor processes. These results align with previously reported musical training-related neural advantages in cerebellar structure observed in both young and older adults who began musical instrument training at an early age (Acer et al., 2018;Gaser & Schlaug, 2003;Hutchinson, 2003;Yamashita et al., 2022). They further supported the musical training effects on cerebellar function, in addition to structural changes.

### 4.4 Musical instrument training effects on WM processing: executive control rather than slave systems

In the cross-sectional comparison, the seed-to-voxel FC analysis revealed lower FC between the left cerebellum IX and the brainstem (pons) during the verbal WM task in the continue group compared with the stop group. Additionally, this lower cerebellum–brainstem FC was associated with better preserved WM. The relevance of the brainstem activation in verbal WM processing has been documented in young adults (Tüdös et al., 2018). PET and fMRI studies have demonstrated the involvement of the brainstem in attention control and its interaction with fronto-parietal cortex (D’Ardenne et al., 2012;Parvizi, 2001;Raizada, 2008;Sturm, 1999). Previous research have provided evidence of musical instrument training effects on the brainstem in auditory processing of speech and music stimuli (Bidelman et al., 2014;Musacchia et al., 2007;Skoe & Kraus, 2012;Wong et al., 2007). It has been suggested that music training initially drives cognitive enhancement in auditory WM, which in turn shapes the corticofugal network (Kraus et al., 2012).

The coupling between the cerebellum and the brainstem during WM task was found to be tighter in never medicated children with attention deficit/hyperactivity disorder (ADHD) compared with healthy controls, suggesting a role of cerebellum–brainstem FC in attention control (Massat et al., 2012). In our study, lower cerebellum–brainstem FC was associated with better preserved verbal WM, indicating better attention control during WM processing in the continue group. This advantage in executive control was further evidenced by the observed group differences in bilateral cerebellar activation during verbal WM tasks. The absence of a lateralization effect in n-back tasks suggested the advantages associated with musical instrument training lie in the enhancement of central executive subfunctions, rather than being confined to subsidiary slave systems only in the phonological loop or sketchpad (Hautzel et al., 2009).

### 4.5 The neural mechanism underlying the musical instrument training effects on verbal WM preservation

A recent review highlighted the potential of Bayesian inference as a fundamental principle for explaining the effects of music-based interventions on aging (Heng et al., 2024). The authors framed aging as an optimization process of Bayesian inference, wherein reliance on consolidated priors increases while the updating of prior models diminishes, linking this phenomenon to cognitive decline in older adults. Conversely, the brain gets trained via processing music as an internal model through Bayesian inference. The review emphasized the role of the striatum in the neuro-endocrine effects of music. Although our study did not directly investigate striatal dopamine metabolism, the observation of better preserved GMV in the right putamen supports the idea that musical training contributes to preserving the structure of the Bayesian brain (Heng et al., 2024). Additionally, the review underscored the cerebellum’s role in rhythmic entrainment, synaptic plasticity, and encoding event likelihood. Our findings of improved cerebellar function among the continuous training group further substantiate the effects of musical training on the Bayesian brain, suggesting that Bayesian inference is a useful framework for explaining how musical training can help prevent age-related cognitive decline.

Our findings are also consistent with the “OPERA” hypothesis, which proposes that the effects of musical training on verbal processing are driven by adaptive plasticity in speech-processing networks when the conditions of Overlap, Precision, Emotion, Repetition, and Attention are met (Patel, 2011). Previous studies have indicated that music and language processing share cognitive resources (Fennell et al., 2021;Schön et al., 2004). The involvement of the right putamen and cerebellum has been demonstrated both in music perception and production (right putamen:Grahn & Rowe, 2009,^2013^;Janata & Grafton, 2003;Kasdan et al., 2022;Lehéricy et al., 2006;Marchand et al., 2008;Raz et al., 2000; cerebellum:Evers, 2023;Evers & Tölgyesi, 2022) and in language processing (right putamen:Copland et al., 2021;Moore et al., 2013;Viñas-Guasch & Wu, 2017; cerebellum:Desmond & Fiez, 1998;Mariën & Borgatti, 2018;Murdoch, 2010;Van Dun et al., 2016). This study demonstrated the neural benefits of musical training as reflected in verbal processing measures, further supporting the OPERA hypothesis from a neuroplasticity perspective.

In summary, this study provides longitudinal evidence supporting the effects of musical training on the Bayesian brain, particularly in the right putamen and bilateral cerebellum, highlighting its potential role in preventing age-related cognitive decline. The correlation between subcortical structure and function with verbal WM outcomes underscores the impact of musical training on verbal processing, illustrating its benefits from a neuroplasticity perspective in healthy older adults.

### 4.6 Contribution and limitations

This study highlighted that musical instrument training-induced neuroplasticity results in less age-related decline among older adults who began musical instrument training later in life, alike older musicians who began musical instrument training in childhood or early adulthood. This finding could encourage older adults to start musical instrument training later in life, as it is never too late to benefit from the participation. Furthermore, the presence of a voluntarily continued group demonstrates that motivation can be sustained over time through learning an instrument, leading to long-term benefits. Unlike tablet-based cognitive training, which may be discontinued, musical instrument training offers a limitless variety of pieces and songs to learn even after the intervention has ended. As such, musical instrument training represents an engaging and enjoyable activity that can sustainably support the successful cognitive aging (Kim & Yoo, 2019;Rogers & Metzler-Baddeley, 2024).

However, this study has several limitations. First, the sample size was relatively small. Participants were recruited from the sample pool of our previous melodica intervention project (Guo et al., 2021). Some older adults dropped out due to personal reasons, such as a health condition making them ineligible for MRI screening, or loss of contact with the welfare center after they stopped participating in group activities there. As a result, only 32 datasets were available, and a healthy/active bias may be introduced among the participants in this study. Additionally, the small sample size could affect the reliability of structural–brain behavior associations, particularly given recent concerns about the replicability of such findings in smaller cohorts. Larger samples are needed in future research to confirm these results (Genon et al., 2022).

Another participant-related limitation was that they were not randomly assigned to either the continuation or stop group; rather, they voluntarily chose to continue melodica practice or to engage in other leisure activities. Motivational factors such as interest in music, a sense of proficiency from mastering the instrument may have contributed to the group differences. Moreover, although there was no statistical difference in age between groups, the continue group was on average 1.85 years older than the stop group. Given that WM capacity has been shown to accelerate its decline after the age of 70 years (Nyberg et al., 2012), the protective effect of musical instrument training on WM might have been underestimated due to the older age of the continue group. Furthermore, since the continue group had only keyboard instrument training experience initiated in older age, it remained to be investigated whether the effects observed can be generalized to musical experience using other instruments or whether different types of musical training might induce distinct effects.

## 5 Conclusion

This study demonstrated that continuous participation in musical instrument training over 4 years in older age led to better preserved verbal WM, GMV in the right putamen, and cerebellar function among healthy older adults. The preservation of verbal WM was correlated with greater cerebellum–pons FC during the verbal WM task in response to increased WM demands. Combined with greater activation during verbal WM processing in the bilateral cerebellum and increased intra-cerebellar FC during visual WM processing in the continue group, these findings suggest an enhanced ability to recruit neural resources in the cerebellum. Furthermore, the decreased cerebral–cerebellar FC indicates a reduced reliance on cortical overactivation, as the cerebellum was sufficiently activated to meet the cognitive demands.

These results highlighted the role of subcortical regions, particularly the putamen and cerebellum, in mediating the musical instrument training effects on cognitive preservation. To our knowledge, this is the first study to provide longitudinal evidence of the causal effects of initiating musical instrument training in older age on mitigating age-related decline in brain and cognition. These findings suggested that it is never too late to start participating in musical instrument training, potentially encouraging older adults to engage in musical instrument training and benefit from its effects.

## Supplementary Material

Supplementary Material

## Data Availability

The individual-level neuroimaging data used in this study are not publicly available. Interested researchers may request access to the data by contacting the corresponding author. Data will be provided upon signing a data sharing agreement and after receiving approval from the requester’s institutional review board (IRB), along with a brief research proposal. The group-level statistical outputs and analysis code will be made available to qualified researchers under similar conditions.
