# Supplementary material for: Never too late to start musical instrument training: Effects on working memory and subcortical preservation in healthy older adults across 4 years
Source: Imaging Neurosci (Camb). 2025 Jun 17;3:IMAG.a.48. doi: 10.1162/IMAG.a.48 (PMC12319798; doi:10.1162/IMAG.a.48)
Supplement: Supplementary Material [file imag.a.48_supp.pdf]

Supplementary material

Supplementary Table 1. Cluster size of each seed for functional connectivity analysis.

	Hemisphere	Volume (mm)
Caudate	Left	6344
	Right	6824
Putamen	Left	7967
	Right	8499
Cerebellum VI	Left	13552
	Right	14360
Cerebellum VII a Crus I	Left	20824
	Right	21184
Cerebellum VII a Crus II	Left	15152
	Right	16936
Cerebellum VII b	Left	4680
	Right	4272
Cerebellum IX	Left	6952
	Right	6472
